# Analysis of Tissue and Serum MicroRNA Expression in Patients with Upper Urinary Tract Urothelial Cancer

**DOI:** 10.1371/journal.pone.0117284

**Published:** 2015-01-28

**Authors:** Stephanie Kriebel, Doris Schmidt, Stefan Holdenrieder, Diane Goltz, Glen Kristiansen, Rudolf Moritz, Christian Fisang, Stefan C. Müller, Jörg Ellinger

**Affiliations:** 1 Universitätsklinikum Bonn, Klinik und Poliklinik für Urologie und Kinderurologie, Bonn, Germany; 2 Universitätsklinikum Bonn, Institut für Klinische Chemie und Klinische Pharmakologie, Bonn, Germany; 3 Universitätsklinikum Bonn, Institut für Pathologie, Bonn, Germany; 4 Universitätsklinikum Münster, Klinik für Urologie, Münster, Germany; Eberhard-Karls University, GERMANY

## Abstract

**Introduction:**

MicroRNAs play an important role in many human malignancies; so far, their expression remains to be studied in upper urinary tract urothelial cancer (UUTUC).

**Materials and Methods:**

The expression of eleven microRNAs (miR-10a, miR-21, miR-96, miR-135, miR-141, miR-182, miR-200b, miR-205, miR-429, miR-520b, miR-1244) formerly shown to be upregulated in urothelial bladder cancer were studied in corresponding normal and cancerous tissue samples of patients undergoing nephroureterectomy for UUTUC. Upregulated microRNAs were then measured in serum samples of patients with UUTUC and patients with non-malignant urological diseases to evaluate their potential as non-invasive biomarkers for UUTUC.

**Results:**

MicroRNA expression allowed differentiation of normal and cancerous tissue: miR-21, miR-96, miR-135, miR-141, miR-182, miR-205, miR-429 and miR-520b were significantly overexpressed. Furthermore, miR-205 was upregulated in poorly differentiated UUTUC. The analysis of circulating RNA in serum demonstrated an increase of miR-141 in patients with UUTUC; receiver operator characteristic analysis demonstrated an area under the curve of 0.726 for miR-141 as a diagnostic biomarker. Furthermore, we observed lower levels of miR-10a and miR-135 in UUTUC patients.

**Conclusions:**

MicroRNA expression is altered in UUTUC. The analysis of circulating miR-141 may be useful to identify patients with UUTUC.

## Introduction

Urothelial carcinoma is one of the most common human malignancies [1]; about 5–10% of these urothelial carcinomas are located in the upper urinary tract (UUTUC, pyelocaliceal cavities and ureter). Although the etiology of UUTUC and bladder cancer is similar, the natural history of UUTUC differs from bladder cancer: UUTUC are often invasive at diagnosis, and thus the prognosis is poorer. Several prognostic factors (i.e. stage, grade, lymph node invasion, lymphovascular invasion, tumor location) have been identified [2]. Still, the prediction of the clinical course of a specific patient remains difficult, as all of the above mentioned factors can only be determined postoperatively. The identification of a preoperative marker, i.e. a serum biomarker, would be helpful to allow better risk stratification [3].

MicroRNAs are small, non-coding RNA molecules, which modify the expression of many human genes and thereby regulate important cellular functions like cell cycle, development, apoptosis and differentiation[4]. Expression profiling studies demonstrated tumor-specific microRNA expression. A recent study demonstrated different microRNA expression profiles in normal renal tissue, UUTUC and renal cell carcinoma, however, UUTUC was not compared to normal urothelial tissue [5]. Given the similarities of UUTUC and urothelial cancer of the bladder, it is likely that microRNA expression is similarly altered. MicroRNAs are detectable in a variety of body fluids including serum [6]. The stability against degradation by RNases and external influences (pH-alteration, storage, freeze/thawing) qualifies circulating microRNAs as non-invasive biomarkers [7,8]. As a consequence, serum microRNAs have been investigated in many malignancies (e.g. bladder cancer [9], kidney cancer [10], prostate cancer [11], testicular cancer [12]).

In order to investigate the role of microRNAs as non-invasive biomarkers in patients with UUTUC, the expression of eleven microRNAs (miR-10a, miR-21, miR-96, miR-135, miR-141, miR-182, miR-200b, miR-205, miR-429, miR-520b, miR-1244) earlier shown to be upregulated in urothelial cancer of the bladder [13–20], was analyzed [11] in corresponding normal ureter and UUTUC tissue. Then, we investigated the serum levels of those target microRNAs with the highest upregulation in patients with UUTUC and patients with non-malignant urological diseases, to explore their diagnostic/prognostic potential.

## Materials and Methods

### 2.1. Sample collection

Written informed consent was obtained from each individual and the study was approved by the local ethics committee (approval number: 036/08 and 049/13). Sample collection was conducted from 2005–2012 (tissue) and 2008–2013 (serum). The clinical-pathological parameters of the study cohort are provided in Table 1.

**Table 1:** Summary of clinical-pathological parameters of the study cohorts.

	tissue cohort		serum cohort	
	cancer n = 47 (%)	control n = 36 (%)	cancer n = 44 (%)	control n = 34 (%)
Age				
mean	68.9	69.1	67.9	63.5
min-max	34–86	34–86	41–88	40–87
Sex				
male	26 (55.3)	22 (61.1)	27 (61.4)	23 (67.6)
female	21 (44.7)	14 (38.9)	17 (38.6)	11 (32.4)
Pathological stage				
pTa	9 (19.1)	n.a.	18 (40.9)	n.a.
pT1	7 (14.9)	n.a.	7 (15.9)	n.a.
pT2	2 (4.3)	n.a.	3 (6.8)	n.a.
pT3	24 (51.1)	n.a.	15 (34.1)	n.a.
pT4	5 (10.6)	n.a.	1 (2.2)	n.a.
lymph node metastasis	2 (4.3)	n.a.	3 (6.8)	n.a.
distant metastasis	8 (17.0)	n.a.	1 (2.2)	n.a.
surgical margins positive	4 (8.5)	n.a.	3 (6.8)	n.a.
vascular invasion	8 (17.0)	n.a.	6 (13.6)	n.a.
lymphovascular invasion	6 (12.8)	n.a.	8 (18.2)	n.a.
Grading				
G1	9 (19.1)	n.a.	6 (13.6)	n.a.
G2	20 (42.6)	n.a.	28 (63.3)	n.a.
G3	18 (38.3)	n.a.	10 (22.7)	n.a.

abbreviations: n.a., not applicable

### 2.2. Tissue samples

MicroRNA levels in cancerous tissue samples from 47 patients undergoing nephroureterectomy at the Department of Urology at the University Hospital Bonn were investigated. Histologically normal ureteral tissue was available from 36 these patients. The samples were fixed in formalin, embedded in paraffin wax and archived in the Institute of Pathology at the University Hospital Bonn.

Using a microtome, 5 to 10 sections (20μm) were produced from each paraffin block. The sections were submerged in heated water and placed onto microscope slides. After drying for 24 hours, the samples were deparaffinized using xylol and dilution series of ethanol. On the basis of haematoxylin-eosin stained sections, on which tumor and normal tissue were marked beforehand, a macro-dissection was done, segregating the tumor and adjacent normal urothelial tissue samples (>1 cm apart).

Total RNA was then extracted using the RecoverAll Total Nucleic Acid Isolation Kit (Life Technologies, Carlsbad, CA, USA) as recommended by the manufacturer. The final elution volume was 50 μL. Quantity and purity of the RNA was determined using the NanoDrop 2000 spectrophotometer (PeqLab, Erlangen, Germany). Reverse transcription was conducted using the miScript II RT Kit (Qiagen, Hilden, Germany) according to manufacturer´s instructions with 400 ng RNA (diluted in a total volume of 12 μL). cDNA was finally diluted at a concentration of 5 ng/μL in HiSpec-Buffer.

### 2.3. Serum samples

We prospectively collected blood samples at the urological departments at the University Hospitals Bonn (n = 10) and Münster (n = 34). The serum samples were handled according to the standard operating procedures of the Biobank initiatives. As a control, an age matched cohort of 34 individuals with non-malignant diseases (benign prostate hyperplasia, urethral stricture, incontinence, urinary stones) undergoing minor urological operations was used. Venous blood was taken in Serum S-Monovette Gel tubes with clotting activator (Sarstedt, Nümbrecht, Germany) prior to surgery. After a minimum of 60 minutes, the clotted serum was centrifuged (10 min, 2500 g), separated and stored in cryotubes at -80°C until use.

Total RNA was isolated from 400 μL serum using the mirVana PARIS Kit (Life Technologies, Carlsbad, CA, USA), after addition of 25 fmol of the synthetic miRNA cel-miR-39 (Qiagen, Hilden, Germany) which allows a quantification of the RNA isolation efficiency. The extraction was performed following the manufacturer´s protocol. Final elution volume was 50μL. Reverse transcription was done using the miScript II RT Kit (Qiagen, Hilden, Germany). Prior to real-time PCR, a preamplification was done in order to gain sufficient material (miScript PreAMP PCR Kit, Qiagen, Hilden, Germany), as preliminary tests showed that serum microRNA levels were too low for direct quantification. The preamplification was done according to the manufacturer´s protocol, using the following cycling conditions: Initial activation step (95°C, 15 minutes), 2-step cycling (denaturation 94°C for 30 seconds, annealing and extension 60°C for 3 minutes) with 12 cycles.

### 2.4. Quantitative real-time PCR

All PCR experiments were conducted in triplicate with 10 μL each on an ABIPrism 7900HT (Life Technologies, Carlsbad, CA, USA) in 384 well plates using the miScript SYBR Green PCR Kit (Qiagen, Hilden, Germany). All PCR primers were purchased from Qiagen. Each run included the cell line RT112 as positive control as well as water, no-RT, TE buffer and genomic DNA as negative controls (preamplified for serum PCR-runs). A serial dilution of RT112 allowed us to measure the PCR amplification efficiency of each PCR-run. The PCR was carried out according to following protocol: 1.Hot Start (95°C, 15 minutes), 2.Denaturation (94°C, 15 seconds), 3.Annealing (55°C, 30 seconds), 4.Extension (70°C, 30 seconds). Each run consisted of 40 cycles of step 2 to 4. Melting curve analysis confirmed the specificity of the PCR products. MicroRNA levels in samples with Cq values >35 were considered as zero; outliers with a Cq difference within triplicates >1 were excluded. PCR data were analyzed with the SDS Relative Quantification Software v2.4 (Life Technologies, Carlsbad, CA, USA) and QBase+ v2.5 (Biogazelle, Zwijnaarde, Belgium). The individual microRNA expression data are provided in the S1 Table (tissue study) and S2 Table (serum study).

MicroRNA expression in tissue was normalized against RNU1–4, SNORD43 and SNORD48. MicroRNA expression in serum was normalized against RNU1–4 and SNORD43, as SNORD48 could not reliably be measured in serum. The suitability of these RNAs as reference gene was demonstrated earlier [21].

### 2.5. Statistical analysis

The Mann-Whitney-U test and the Kruskal-Wallis-test were used to evaluate differences between cancer patients and healthy controls and to associate microRNA expression with clinical-pathological parameters, as appropriate. Receiver Operator Curve (ROC) analyses were performed to determine the sensitivity and specificity of microRNAs as diagnostic biomarkers; the Youden index was used to identify the optimal threshold. Statistical significance was concluded at p<0.05, and Bonferroni correction for multiple hypothesis testing was performed (tissue experiments p<0.0045, serum experiments p<0.0063). Unsupervised two-way hierarchical cluster analysis with Euclidian distance was performed to classify tumor and normal urothelial tissue samples. Statistical analyses were performed using SPSS Statistics v21 (IBM, Chicago, Illinois, USA).

## Results

### 3.1 Analysis of microRNA expression in upper urinary tract urothelial cancer tissue

**Fig 1:**
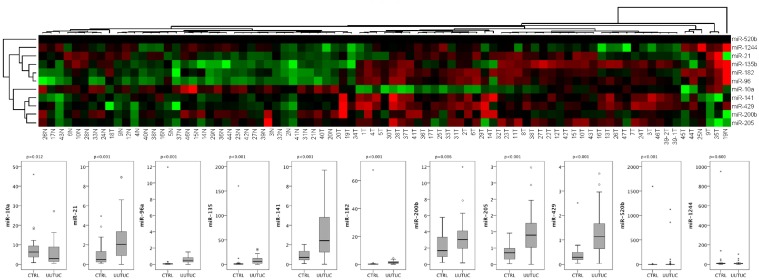
Unsupervised two-way hierarchical cluster analysis with Euclidian distance was performed to classify normal and cancerous urothelial tissue samples. The log2-fold-change was used to construct the heatmap which indicates that a microRNA expression profiling allows differentiation of normal ureter and urothelial carcinoma tissue. The samples from normal urothelial tissue are coded with “N”, the cancer samples with “T”. The Boxplot diagrams indicate relative levels of each target microRNA.

MicroRNA expression has not been investigated in UUTUC tissue so far. We thus determined the expression of eleven microRNAs earlier shown to be overexpressed in urothelial bladder cancer in 47 UUTUC tissue samples and 36 corresponding samples of histologically normal ureters. We observed a significant overexpression of miR-21, miR-96, miR-135, miR-141, miR-182, miR-205, miR-429, miR-520b (all p<0.001) in UUTUC; the microRNAs miR-10a (p = 0.012) and miR-200b (p = 0.006) showed a distinct trend towards upregulation, whereas miR-1244 (p = 0.600) was similar in normal and malignant tissue. miR-205 (p = 0.002) was upregulated in undifferentiated UUTUC (G3 vs. G2/G1). See Fig. 1.

### 3.2 Analysis of circulating serum microRNAs in upper urinary tract cancer patients

**Fig 2:**
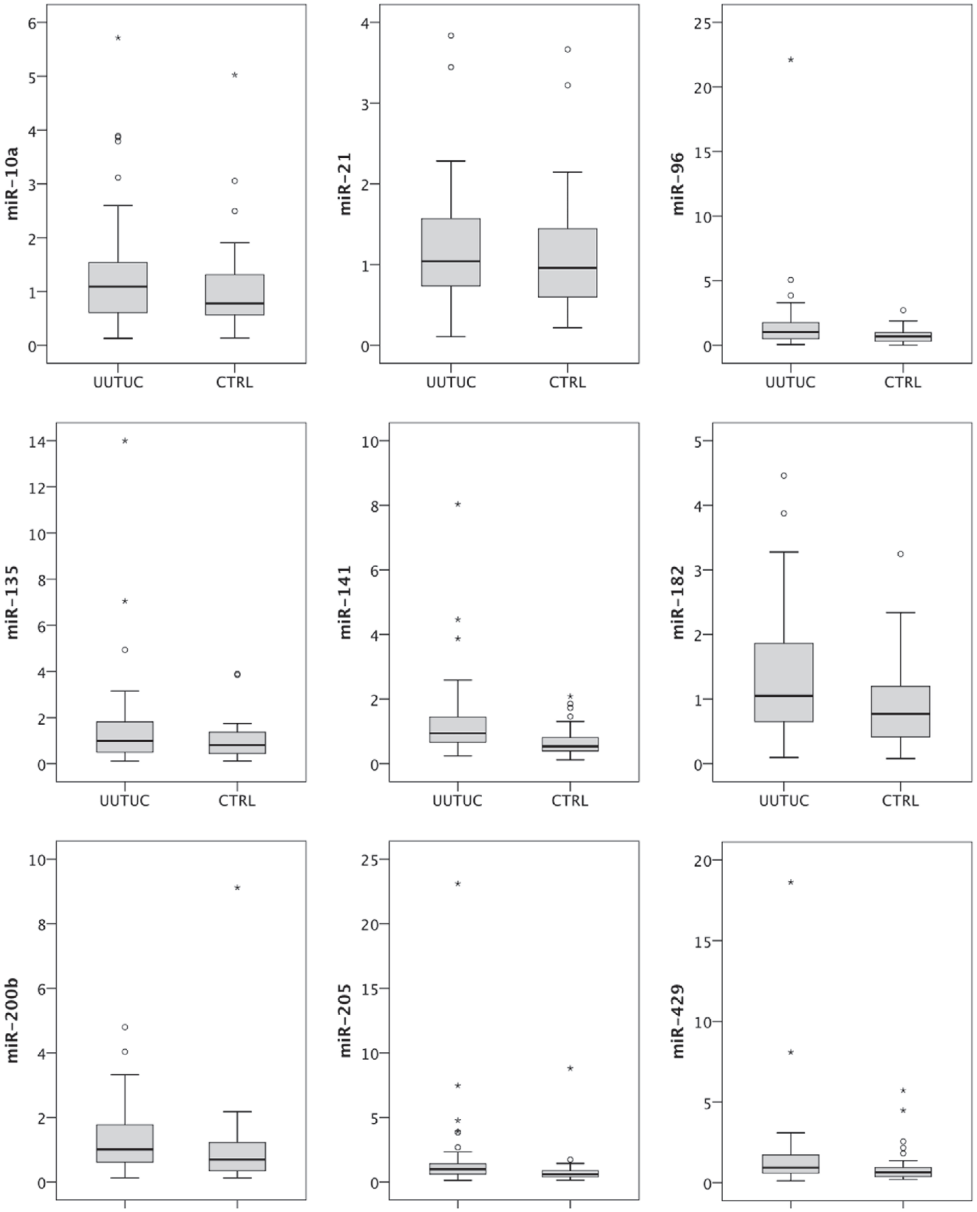
Serum microRNA levels are increased in patients with upper urinary tract urothelial carcinoma compared to patients with non-malignant disease. The Mann-Whitney-U test was used to evaluate differences between the groups.

**Fig 3:**
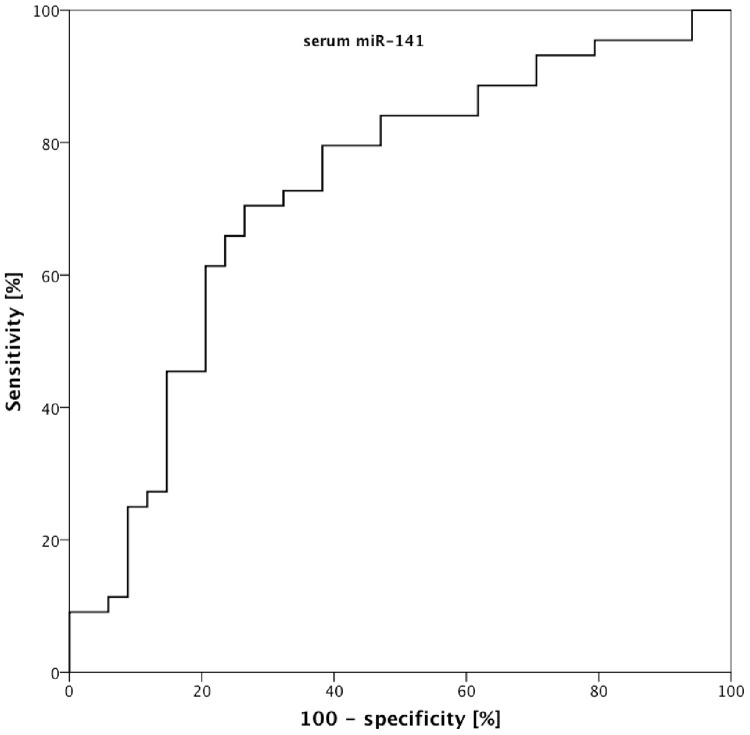
Receiver operator characteristic analysis demonstrates that serum microRNA miR-141 levels allow sensitive (70.5%) and specific (73.5%) discrimination of patients with upper urinary tract urothelial carcinoma and control subjects.

We next investigated serum levels of the candidate microRNAs identified during tissue profiling: serum samples of patients with UUTUC (n = 44) in comparison to patients with non-malignant urological diseases (n = 34) treated at two tertiary referral clinics were investigated. The microRNA expression levels in serum are displayed in Fig. 2. miR-141 was circulating at significantly higher levels (p<0.001) in serum of UUTUC patients than in control subjects (mean 1.30 vs. 0.69). ROC analysis demonstrated an AUC of 0.726 (95% confidence interval 0.609–0.843) for miR-141 as diagnostic biomarker; see Fig. 3 and Table 2. miR-200b (p = 0.041), miR-205 (p = 0.008), miR-425 (p = 0.025), miR-96 (0.013) showed a trend towards higher levels in cancer patients, but the p-value was >0.0065 which was defined as significance level after correction for multiple hypothesis testing. miR-182 (p = 0.083), miR-21 (p = 0.532) and miR-135 (p = 0.261) were similar in UUTUC patients and controls.

**Table 2:** Receiver operator characteristic analysis for serum microRNAs.

	AUC (95%CI)	Cut-off	Sensitivity (%)	Specificity (%)
miR-10a	0.560 (0.432–0.689)	1.25	43.2	73.5
miR-21	0.541 (0.410–0.672)	0.49	84.1	29.4
miR-96	0.575 (0.447–0.702)	0.82	63.6	67.6
miR-135	0.575 (0.447–0.702)	1.81	29.5	91.2
miR-141	0.726 (0.609–0.843)	0.77	70.5	73.5
miR-182	0.615 (0.489–0.741)	0.87	63.6	61.8
miR-200b	0.635 (0.511–0.759)	1.49	70.5	55.9
miR-205	0.675 (0.555–0.795)	1.01	50.0	85.3
miR-429	0.649 (0.525–0.774)	0.74	65.9	61.8

abbreviations: 95%CI, 95% confidence interval

Then, we correlated serum microRNA levels with clinical-pathological parameters. Serum miR-10a (p = 0.003) was decreased in muscle-invasive UUTUC (pTa/pT1:1.79 vs. pT2–4: 0.79). miR-135 also showed a trend towards lower levels in cancer patients with muscle-invasive tumors (2.18 vs. 0.96; p = 0.040). We did not observe a correlation with age, sex, metastasis or grading (all p>0.05) and circulating microRNA levels.

## Discussion

The interest in microRNA increased dramatically during the past years, and tissue and tumor-specific expression profiles have been characterized. Given the similarities of urothelial cancer of the bladder and the upper urinary tract, we performed a literature search to identify a set of dysregulated microRNAs in bladder cancer [13–19] which are of interest for expression profiling in UUTUC tissue and serum samples. Very recently, tissue microRNA expression profiles in UUTUC have been published [5,22]: however, the number of UUTUC samples (n = 5) was very small and not compared to normal urothelial tissue[5], or the study was restricted to malignant samples [22].

We first observed that microRNAs expression profiles allowed distinguishing cancerous and normal ureter/renal pelvis samples with high accuracy. Especially miR-21, miR-96, miR-135, miR-141, miR-182, miR-205, miR-429 and miR-520b were distinctly overexpressed in UUTUC. Interestingly, this partly overlaps with the microRNA expression profile earlier established in a prostate cancer study, in which miR-205, miR-96 and miR-182 were found particularly regulated [23]. We could also confirm the similar molecular carcinogenesis of urothelial cancer of the bladder and the upper urinary tract as upregulation of many microRNAs earlier identified as oncomirs in bladder cancer were also seen in UUTUC[13–19]. Recently, Zaravinos et al. [20] investigated microRNA expression in normal renal tissue and renal tumors (renal cell carcinoma and UUTUC): 21 microRNAs were specifically deregulated in UUTUC (5 microRNAs upregulated and 16 microRNAs downregulated—the UUTUC profile was different from our target microRNAs); miR-10a, miR-200b and miR-205 were upregulated compared to renal tissue. It should be noted that the lack of normal urothelial tissue as appropriate control limits the informative value (urothelium specific expression is not controlled) [20].

We next investigated the serum levels of these microRNAs in a two-center-cohort of patients with UUTUC and non-malignant diseases. We observed a significant increase of miR-141 in UUTUC patients: mean levels were almost twice as high compared to controls; several other candidates (miR-200b, miR-205, miR-425 and miR-96) also showed a trend towards increased levels in UUTUC patients. ROC analyses demonstrated that miR-141 allowed distinguishing UUTUC patients and controls with an AUC of 0.726 (sensitivity 71% and specificity 74%) indicating that circulating miR-141 is a suitable diagnostic biomarker. To the best of our knowledge, this is the first description of a nucleic acid biomarker in blood of patients with UUTUC. In an earlier study on patients with urothelial bladder cancer, a significant increase of miR-141 in serum of cancer patients was not observed, although a trend towards higher miR-141 levels compared to control subjects could be seen[9]. Stage and grade could explain this difference: high serum miR-141 levels were observed in many studies in advanced stages [24,25] or patients with recurrence after surgery [25–27]; however, miR-141 was not correlated with any prognostic parameter in our study on UUTUC. The finding of increased urinary miR-141 levels in bladder cancer patients supports the idea of miR-141 as a diagnostic tool [28]. It should be noted that circulating miR-141 levels are elevated in multiple tumor entities including prostate cancer [25], colon cancer [29] and cervical cancer [30]. An overexpression of miR-141 is involved in KEAP1-regulated cisplatin resistance [31], and thus miR-141 measurements could be helpful for the monitoring of patients undergoing cisplatin chemotherapy for metastatic UUTUC. The TarBase 6.0 [32], a database with experimentally validated microRNA-gene interactions, indicates that miR-141 regulates various genes (for example PTEN [33], ERBB2IP [34], HOXB5 [34], E2F3 [33], cyclin D1 [33]) involved in urothelial carcinogenesis (database query: 06–03–2014).

The miR-205 tissue levels were also correlated with undifferentiated UUTUC, and miR-10a and miR-135 were decreased in serum of patients with muscle-invasive UUTUC. Tumor grade and stage are usually correlated in urothelial cancer, and thus one would expect significant correlations with stage (miR-205) or grade (miR-10a, miR-135), respectively. Several explanations are possible: *(i)* certain biological processes induce microRNA alterations in later stages of carcinogenesis; (*ii*) three (out of ten) patients with non-muscle invasive disease had G3 tumors, and thereby limit the correlation of stage and grade; and (*iii*) a finding by chance may not be excluded. Thus, the analysis of microRNAs could be useful for prognostic purposes, although future studies are required to confirm our results. Notably, miR-205 [35] and miR-10a [36] were associated with the survival time in patients with bladder cancer. The recent report by Izquierdo et al. indicates that miR-149 levels in tumor tissue are predictive of cancer-specific survival in patients with UUTUC [22]; an analysis of this microRNA in serum in a future study would be of interest. However, miR-149 was not detectable in serum of neither a patient with prostate cancer nor a healthy subject, even though a preamplification step was employed according to Mitchell et al. [37], concluding that miR-149 levels in blood samples are may be too low to detect.

A novel biomarker has to prove itself against established markers; urine cytology in patients suffers from a poor sensitivity [38], even in patients with high grade UUTUC. The inclusion of conjunctive markers (BTA stat test [39] or FISH [40]) approaches may increase the diagnostic information significantly, but low-grade cancer remain difficult to detect. The performance of serum miR-141 seems to be somewhat lower compared to urinary markers (sensitivity 71%, specificity 74%), but similar detection rates in muscle-invasive and non-muscle invasive UUTUC justify future research to explore its value.

Some limitations should be acknowledged: we compared microRNA expression in cancerous and adjacent normal tissue. Although the normal tissue was histologically normal and located at least 1 cm distant from the tumor, the so-called “field effect” could cause molecular alterations in this control tissue. However, the comparison of malignant and normal tissue from the same patients minimizes the effect of interindividual microRNA expression differences. The sample size in our study was only moderate (47 cancer vs. 36 normal tissues; and 44 UUTUC vs. 34 control serum samples), but the relative rarity of UUTUC compared to bladder cancer or renal cell carcinoma make the analysis of large cohorts difficult.

## Conclusions

MicroRNA expression is dysregulated in UUTUC tissue; these changes lead to altered microRNA profiles in patients’ circulation. The detection of increased miR-141 levels allows identification of patients with UUTUC; if future studies confirm this finding, measurement of serum microRNAs may help clinicians to manage these patients.

## Supporting Information

S1 Table Summary of tissue microRNA expression levels in patients with upper urinary tract urothelial cancer. (XLSX)

S2 Table Summary of serum microRNA expression levels in patients with upper urinary tract urothelial cancer. (XLSX)
